# Employing nanopore sequencing on FFPE-derived DNA for CNS tumor diagnostics

**DOI:** 10.1186/s40478-025-02172-z

**Published:** 2025-11-06

**Authors:** Paul Kerbs, Michelle Brehm, Daniel Haag, Henri Bogumil, Areeba Patel, Martin Sill, Natalie Berghaus, Kirsten Göbel, Daniel Schrimpf, Ghazaleh Tabatabai, Jens Schittenhelm, Oliver Sakowitz, Christine Stadelmann, Roland Goldbrunner, Nima Etminan, Miriam Ratliff, Christel Herold-Mende, Sandro Krieg, Wolfgang Wick, David T. W. Jones, Andreas von Deimling, Felix Sahm, Pauline Göller

**Affiliations:** 1 Department of Neuropathology, University Hospital Heidelberg, Heidelberg, Germany; 2 German Consortium for Translational Cancer Research (DKTK), German Cancer Research Center (DKFZ), Heidelberg, Germany; 3 Division of Pediatric Glioma Research, Hopp Children’s Cancer Center (KiTZ), Heidelberg, Germany; 4 Department of Pathology, Institute of Pathology and Neuropathology, Tübingen, Germany; 5 Department of Neurosurgery, Neurosurgery Center Ludwigsburg-Heilbronn, Ludwigsburg, Germany; 6 Department of Neuropathology, University Medical Center Göttingen, Göttingen, Germany; 7 Department of General Neurosurgery, Center for Neurosurgery, University Hospital of Cologne, Cologne, Germany; 8 Department of Neurosurgery, University Hospital Mannheim, Mannheim, Germany; 9 Department of Neurosurgery, University Hospital Heidelberg, Heidelberg, Germany; 10 National Center for Tumor Diseases (NCT), NCT Heidelberg, a partnership between DKFZ and Heidelberg University Hospital, Heidelberg, Germany

The incorporation of methylation analyses into diagnostic neuropathology has significantly improved the precision of tumor classification and informed treatment decisions [1]. Despite these advances, challenges such as higher costs, specialized equipment requirements, and extended turnaround times persist. Oxford Nanopore Technologies (ONT) sequencing provides a unique solution for rapid epigenetic profiling using cost-effective instruments. Notably, ONT sequencing has enabled methylation-based classification of central nervous system (CNS) tumors from fresh-frozen samples within just 30 min of sequencing [2]. This technology has also been applied to formalin-fixed paraffin-embedded (FFPE) tissue samples [3], overcoming challenges posed by lower DNA quality and fragmentation. However, the minimum sequencing duration for accurate classification and the method’s performance across various CNS tumor types in FFPE samples require further investigation.

To address these gaps, we evaluated CNS tumor classification across various sequencing time points and systematically assessed copy number variation (CNV) profiling in a more diverse cohort of FFPE samples. Additionally, we investigated the impact of different DNA extraction protocols on CpG site coverage.

For this study, we selected a cohort of archival FFPE samples from 40 diverse CNS tumors (Fig. 1A, Supplementary Table 1) including: glioblastoma IDH-wildtype WHO grade 4 (n = 8), astrocytoma IDH-mutant (WHO grades 2–4, n = 8), meningioma (WHO grades 1–3, n = 6), oligodendroglioma IDH-mutant and 1p/19q-codeleted (WHO grades 2–3, n = 4), pilocytic astrocytoma WHO grade 1 (n = 2), ependymal tumors (n = 3), medulloblastoma WHO grade 4 (n = 2), central neurocytoma WHO grade 2 (n = 2), diffuse midline glioma H3 K27-altered WHO grade 4 (n = 1), atypical choroid plexus papilloma WHO grade 2 (n = 1), peripheral nerve sheath tumor (n = 1), CNS sarcoma (n = 1), and atypical teratoid/rhabdoid tumor WHO grade 4 (n = 1). DNA was extracted using the Maxwell RSC FFPE DNA purification kit (Promega, AS1720). Sequencing libraries were prepared from 2–3 µg of input DNA using the ligation sequencing kit (Oxford Nanopore Technologies, SQK-LSK114) with minor adjustments to the protocol [4]. Sequencing was performed on FLO-PRO114M flow cells for 24 h. Raw data were basecalled using Dorado v0.6.3 (basecall model v4.3.0) and mapped to the T2T-CHM13v2.0 reference genome, wherein canonical or modified cytosine bases at CpG sites were extracted with Modkit v0.2.4 using a probability threshold of 0.85. Methylation-based tumor classification was performed using three distinct algorithms: the random forest classifier from Rapid-CNS^2^ [5], the neural network-based classifier from nanoDx [6] and MethyLYZR [7], which employs a naïve Bayesian framework. These classifications were benchmarked against the reference Heidelberg classifier [1] using matched Illumina methylation array data. The results from the Heidelberg classifier are hereafter referred to as “EPIC”.

**Fig. 1:**
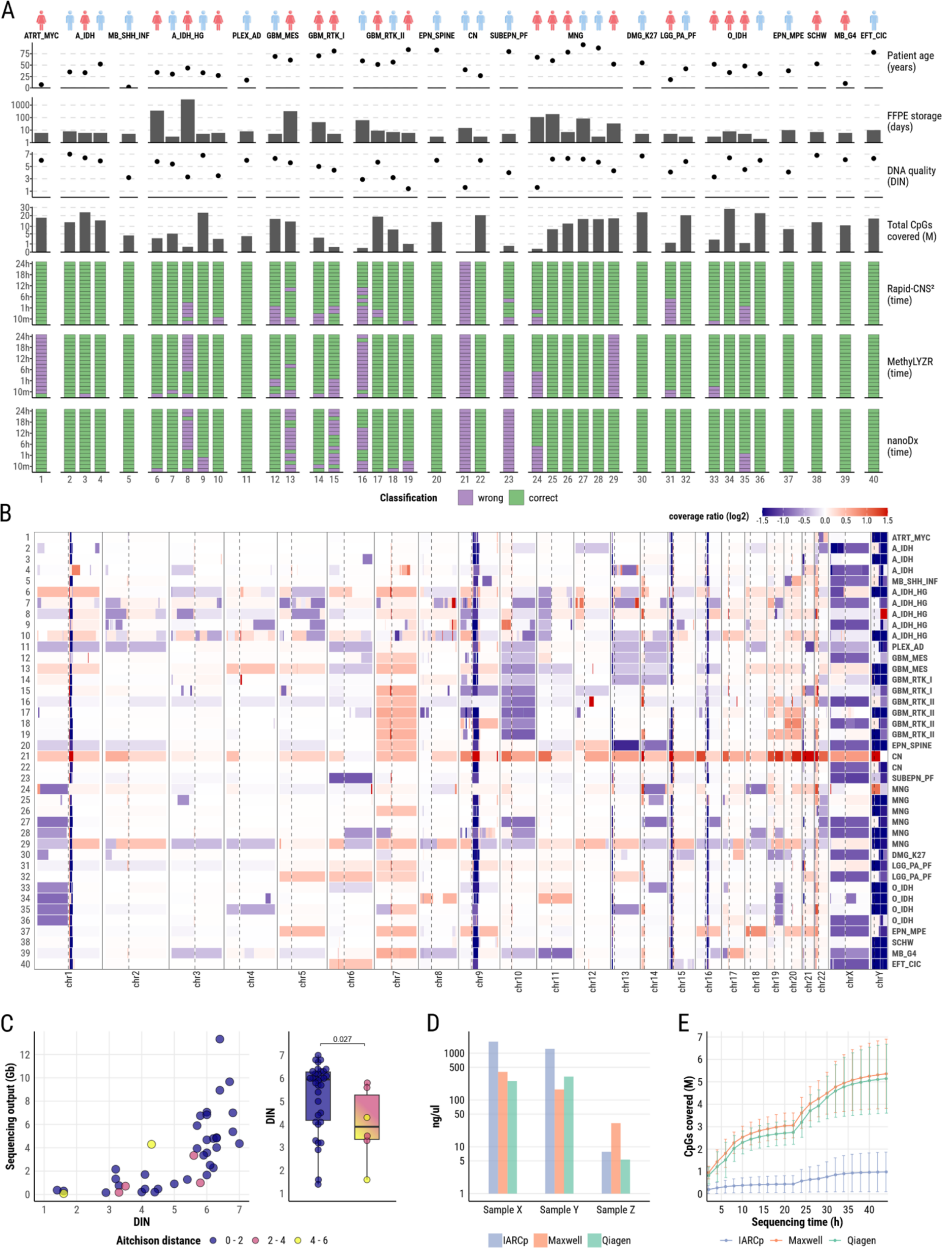
**A** Overview and summary of methylation analysis results. **B** CNV profiles generated from ONT sequencing of FFPE samples. **C** Concordance of CNV profiles from ONT sequencing of FFPE samples compared to profiles derived from matched methylation array data, quantified using the Aitchison distance. **D** Average DNA yield (6 technical replicates) using different extraction protocols. **E** Impact of different DNA extraction protocols on captured CpG sites from Sample X and Y over sequencing time

The raw sequencing output showed considerable variability, with an average of 2.99 Gb (SD = 2.94) per tumor sample, corresponding to an average genome coverage of 0.83x (SD = 0.83). The generated sequences covered on average 10.7 million CpG sites (SD = 8.5). After 24 h of sequencing, Rapid-CNS^2^, MethyLYZR, and nanoDx achieved concordant classification with EPIC in 39 of 40, 35 of 40, and 35 of 40 cases, respectively. “Robust classification”, determined independently for each classifier, was defined as the earliest sequencing time point after which results stabilized and aligned with EPIC. To illustrate high prediction variability observed in the cohort, detailed results for representative samples 8 and 16 are shown in Supplementary Figs. S1-S2. Rapid-CNS^2^, MethyLYZR, and nanoDx robustly classified 75% of samples after 60, 40, and 20 min, respectively (Supplementary Fig. S3). Samples requiring extended sequencing times to achieve robust classification exhibited significantly lower DNA Integrity Numbers (DIN; Supplementary Figs. S4-S6). One sample, misclassified by all classifiers, had particularly poor quality (DIN = 1.6), resulting in low sequencing output and insufficient CpG site capture. In three cases, MethyLYZR identified closely related but discordant subtypes compared to EPIC: ATRT_MYC as ATRT_SHH, A_IDH_HG as A_IDH, and GBM_RTK_II as GBM_MES. In one case, MethyLYZR reported SFT_HMPC in contrast to MNG as predicted by EPIC. Integrated diagnosis based on data from routine diagnostics supported the results obtained from EPIC. In four cases, nanoDx identified closely related but discordant subtypes compared to EPIC: A_IDH_HG as A_IDH, GBM_MES as GBM_RTK_II, GBM_RTK_I as GBM_RTK_II, and SUBEPN_PF as EPN_PF_B. Additionally, we computed CNV profiles using CNVkit [8] v0.9.10, with the binsize parameter set to 200,000, while excluding low-coverage regions and centromeres (Fig. 1B). We systematically compared the results to profiles previously generated from matched methylation array data by summarizing segmental CNV calls as ratios per chromosomal arm and employing Aitchison distance (AD) metric [9]. AD values were averaged per sample (Fig. 1C) with a median of 0.97 across samples, demonstrating high overall concordance. Samples with AD ≥ 2 exhibited significantly lower DIN values (median = 3.9, n = 6) compared to samples with AD < 2 (median = 5.95, n = 34; one-sided, unpaired Wilcoxon rank-sum test, *p* = 0.027). Despite the variation in AD values, visual inspection of the profiles revealed a high degree of similarity even for samples with AD $\ge$ 2, as illustrated in Supplementary Figs. S7–S12.

To evaluate the impact of FFPE DNA extraction methods on CNS tumor methylation profiling, we additionally tested two alternative protocols, namely the QIAamp DNA FFPE tissue kit (Qiagen, 56,404) and the IARCp protocol [10]. DNA from three different FFPE tumor samples was extracted in duplicates and quantity measurements were performed in triplicates (Fig. 1D, Supplementary Fig. S13). While we observed comparable DNA yields between Maxwell and Qiagen across samples, the IARC protocol demonstrated notably higher yields in 2 out of 3 samples. Libraries were prepared from the two samples showing the highest DNA yield (Sample X, Y) as described above and sequenced for 44 h. While IARCp yielded greater DNA quantities, we noted that DNA extracted by Maxwell allowed for covering the highest number of CpG sites, presenting Maxwell as the superior protocol for this task (Fig. 1E).

In summary, our study on a diverse cohort of FFPE samples, encompassing a wide range of CNS tumor subtypes, demonstrates that ONT sequencing enables precise tumor methylation classification within 24 h, alongside accurate CNV profiling that exhibits high concordance with standard methylation array data. Robust classification, previously achieved within minutes for fresh-frozen samples [2, 11], is also attainable with FFPE-derived DNA. However, classification efficiency depends on the quality of extracted DNA, with lower-quality samples requiring extended sequencing times and increasing the risk of misclassification. We propose that ONT sequencing represents a cost-effective technology for rapid CNS tumor classification across diverse sample types, enhancing patient care through its speed, accuracy, and affordability.

## Supplementary Information

Supplementary Material 1

## Data Availability

The datasets used and/or analyzed during the current study are available from the corresponding author on reasonable request.

## Abbreviations

AD: Aitchison distance
A_IDH_HG: Astrocytoma IDH-mutant high-grade
A_IDH: Astrocytoma IDH-mutant low-grade
ATRT_MYC: Atypical teratoid/rhabdoid tumor, subclass MYC
ATRT_SHH: Atypical teratoid/rhabdoid tumor, subclass SHH
CN: Central neurocytoma
CNS: Central nervous system
CNV: Copy number variation
DMG_K27: Diffuse midline glioma H3 K27-altered
EFT_CIC: CNS Ewing sarcoma family tumor with CIC alteration
EPN_MPE: Ependymoma, myxopapillary
EPN_SPINE: Ependymoma, spinal
FFPE: Formalin-fixed paraffin-embedded
GBM_RTK_I: Glioblastoma multiform, subclass RTKI
GBM_RTK_II: Glioblastoma multiform, subclass RTKII
GBM_MES: Glioblastoma multiform, subclass mesenchymal
LGG_PA_PF: Low-grade glioma, subclass midline pilocytic astrocytoma
MB_G4: Medulloblastoma, subclass group 4
MB_SSH_INF: Medulloblastoma, subclass SHH infant
MNG: Meningioma
ONT: Oxford Nanopore Technologies
O_IDH: Oligodendroglioma, IDH-mutant
PLEX_AD: Plexus tumor, subclass adult
SCHW: Schwannoma
SFT_HMPC: Solitary fibrous tumor/hemangiopericytoma
SUBEPN_PF: Subependymoma, posterior fossa
