# Supplementary material for: Employing nanopore sequencing on FFPE-derived DNA for CNS tumor diagnostics
Source: Acta Neuropathol Commun. 2025 Nov 6;13:226. doi: 10.1186/s40478-025-02172-z (PMC12590785; doi:10.1186/s40478-025-02172-z)
Supplement: Supplementary file 1 — Supplementary Material 1 [file 40478_2025_2172_MOESM1_ESM.docx]

# Supplementary Materials

## Employing Nanopore-Sequencing on FFPE-derived DNA for CNS tumor diagnostics

Paul Kerbs, Michelle Brehm, Daniel Haag, Henri Bogumil, Areeba Patel, Martin Sill, Natalie Berghaus, Kirsten Göbel, Daniel Schrimpf, Ghazaleh Tabatabai, Jens Schittenhelm, Oliver Sakowitz, Christine Stadelmann-Nessler, Roland Goldbrunner, Nima Etminan, Miriam Ratliff, Christel Herold-Mende, Sandro Krieg, Wolfgang Wick, David T.W. Jones, Andreas von Deimling, Felix Sahm, Pauline Göller

Table of Contents

[**Table 1:** Details of the FPPE sample cohort. 1](#_Toc202179491)

[**Figure S1:** Detailed classification results over sequencing time for sample 8. 2](#_Toc211683599)

[**Figure S2:** Detailed classification results over sequencing time for sample 16. 2](#_Toc211683600)

[**Figure S3:** Summary of robustly classified samples across sequencing time points. 3](#_Toc211683601)

[**Figure S4:** DIN values compared between robustly and non-robustly classified samples by Rapid-CNS^2^ across sequencing time points. 4](#_Toc211683602)

[**Figure S5:** DIN values compared between robustly and non-robustly classified samples by MethyLYZR across sequencing time points. 4](#_Toc211683603)

[**Figure S6:** DIN values compared between robustly and non-robustly classified samples by nanoDx across sequencing time points. 5](#_Toc211683604)

[**Figure S7:** Visual comparison of the CNV profile for sample 6. 6](#_Toc211683605)

[**Figure S8:** Visual comparison of the CNV profile for sample 8. 7](#_Toc211683606)

[**Figure S9:** Visual comparison of the CNV profile for sample 10. 8](#_Toc211683607)

[**Figure S10:** Visual comparison of the CNV profile for sample 13. 9](#_Toc211683608)

[**Figure S11:** Visual comparison of the CNV profile for sample 21. 10](#_Toc211683609)

[**Figure S12:** Visual comparison of the CNV profile for sample 29. 11](#_Toc211683610)

[**Figure S13:** Statistical comparison of DNA yields across extraction protocols. 12](#_Toc211683611)

**Table 1:** Clinical details of the patients, sequencing and quality metrics of the respective FPPE samples.

| **Sample ID** | **Sex** | **Patient age (years)** | **Methylation class** | **FFPE storage (days)** | **DIN** | **Sequencing output (Gb)** | **Total CpGs covered (M)** | **Genome coverage** |
| --- | --- | --- | --- | --- | --- | --- | --- | --- |
| 1 | f | 7.14 | ATRT_MYC | 6 | 6.0 | 4.264 | 17.794 | 1.160 |
| 2 | m | 35.06 | A_IDH | 8 | 7.0 | 3.504 | 13.362 | 0.960 |
| 3 | f | 33.46 | A_IDH | 6 | 6.4 | 8.385 | 23.785 | 2.260 |
| 4 | m | 52.39 | A_IDH | 6 | 5.9 | 3.025 | 15.075 | 0.870 |
| 5 | m | 1.71 | MB_SHH_INF | 5 | 3.2 | 1.024 | 4.164 | 0.260 |
| 6 | f | 34.04 | A_IDH_HG | 358 | 5.8 | 0.598 | 2.869 | 0.160 |
| 7 | m | 30.36 | A_IDH_HG | 3 | 5.4 | 1.076 | 5.093 | 0.300 |
| 8 | f | 43.61 | A_IDH_HG | 2808 | 3.3 | 0.067 | 0.418 | 0.020 |
| 9 | m | 33.25 | A_IDH_HG | 5 | 6.8 | 6.311 | 23.315 | 1.860 |
| 10 | f | 27.28 | A_IDH_HG | 6 | 3.5 | 0.492 | 2.625 | 0.130 |
| 11 | m | 17.11 | PLEX_AD | 8 | 6.0 | 1.066 | 3.816 | 0.270 |
| 12 | m | 69.15 | GBM_MES | 5 | 6.3 | 4.176 | 16.547 | 1.270 |
| 13 | f | 61.09 | GBM_MES | 321 | 5.6 | 2.884 | 14.074 | 0.840 |
| 14 | f | 70.42 | GBM_RTK_I | 43 | 5 | 0.618 | 3.110 | 0.200 |
| 15 | f | 81.03 | GBM_RTK_I | 5 | 4.4 | 0.067 | 0.389 | 0.020 |
| 16 | m | 59.40 | GBM_RTK_II | 62 | 2.9 | 0.046 | 0.254 | 0.010 |
| 17 | f | 51.52 | GBM_RTK_II | 9 | 5.7 | 5.479 | 18.954 | 1.460 |
| 18 | m | 56.64 | GBM_RTK_II | 7 | 3.2 | 1.771 | 7.645 | 0.450 |
| 19 | f | 84.01 | GBM_RTK_II | 6 | 1.4 | 0.208 | 0.990 | 0.050 |
| 20 | m | 83.05 | EPN_SPINE | 3 | 6.0 | 3.189 | 13.586 | 0.870 |
| 21 | m | 39.85 | CN | 15 | 1.6 | 0.002 | 0.003 | 0.000 |
| 22 | m | 26.81 | CN | 3 | 6.0 | 6.578 | 20.524 | 1.710 |
| 23 | m | 79.74 | SUBEPN_PF | 5 | 4.0 | 0.090 | 0.567 | 0.020 |
| 24 | f | 67.24 | MNG | 110 | 1.6 | 0.065 | 0.162 | 0.020 |
| 25 | f | 59.87 | MNG | 190 | 6.2 | 1.773 | 7.868 | 0.470 |
| 26 | f | 78.51 | MNG | 7 | 6.3 | 3.476 | 12.211 | 0.930 |
| 27 | m | 94.81 | MNG | 84 | 6.2 | 4.351 | 16.457 | 1.150 |
| 28 | m | 87.63 | MNG | 3 | 5.7 | 3.603 | 16.371 | 1.030 |
| 29 | f | 52.20 | MNG | 34 | 4.3 | 3.916 | 17.120 | 1.160 |
| 30 | m | 55.08 | DMG_K27 | 5 | 6.7 | 9.212 | 23.889 | 2.540 |
| 31 | f | 18.21 | LGG_PA_PF | 5 | 4.1 | 0.240 | 1.300 | 0.060 |
| 32 | m | 42.05 | LGG_PA_PF | 3 | 5.8 | 6.295 | 20.439 | 1.720 |
| 33 | f | 51.98 | O_IDH | 3 | 3.3 | 0.481 | 2.318 | 0.120 |
| 34 | f | 33.77 | O_IDH | 8 | 6.4 | 12.530 | 28.164 | 3.630 |
| 35 | f | 48.01 | O_IDH | 5 | 4.5 | 0.204 | 1.211 | 0.050 |
| 36 | m | 31.30 | O_IDH | 2 | 6.0 | 6.201 | 22.819 | 1.820 |
| 37 | m | 37.61 | EPN_MPE | 10 | 4.1 | 1.438 | 7.977 | 0.400 |
| 38 | f | 52.90 | SCHW | 7 | 6.8 | 4.516 | 13.359 | 1.190 |
| 39 | f | 9.80 | MB_G4 | 6 | 6.1 | 2.172 | 10.695 | 0.590 |
| 40 | m | 78.16 | EFT_CIC | 10 | 6.3 | 4.149 | 16.916 | 1.160 |


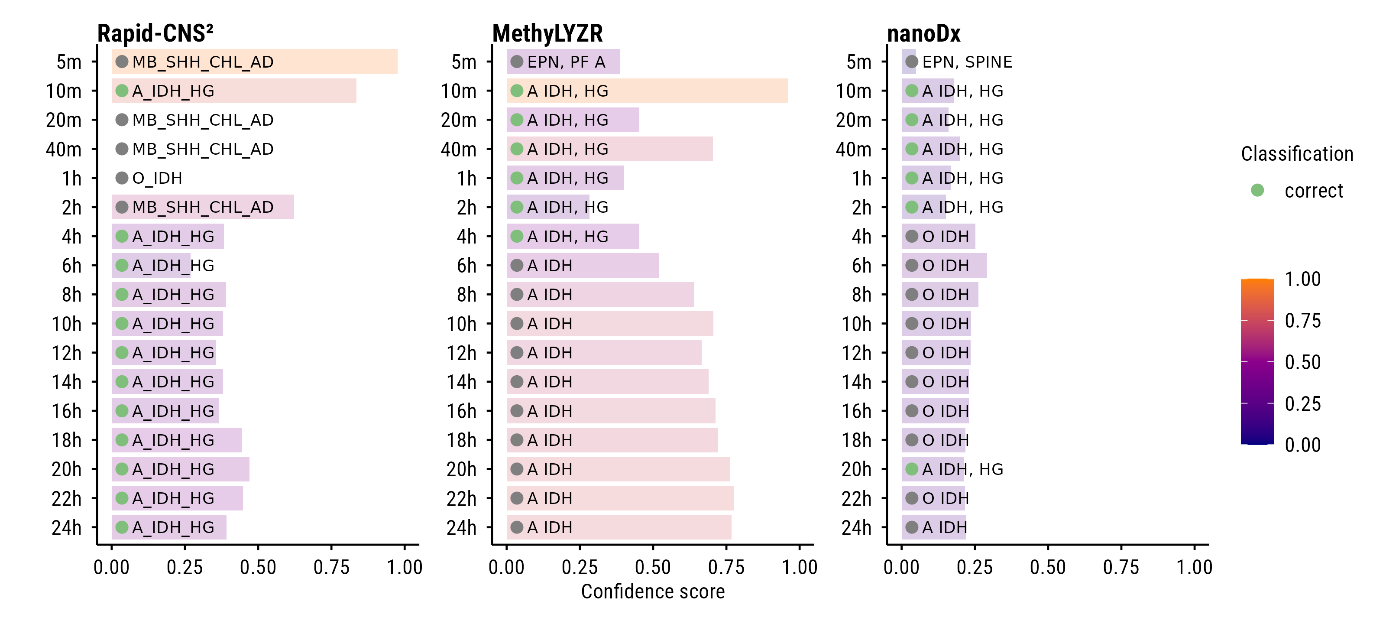


**Figure S1:** Detailed classification results over sequencing time for sample 8.


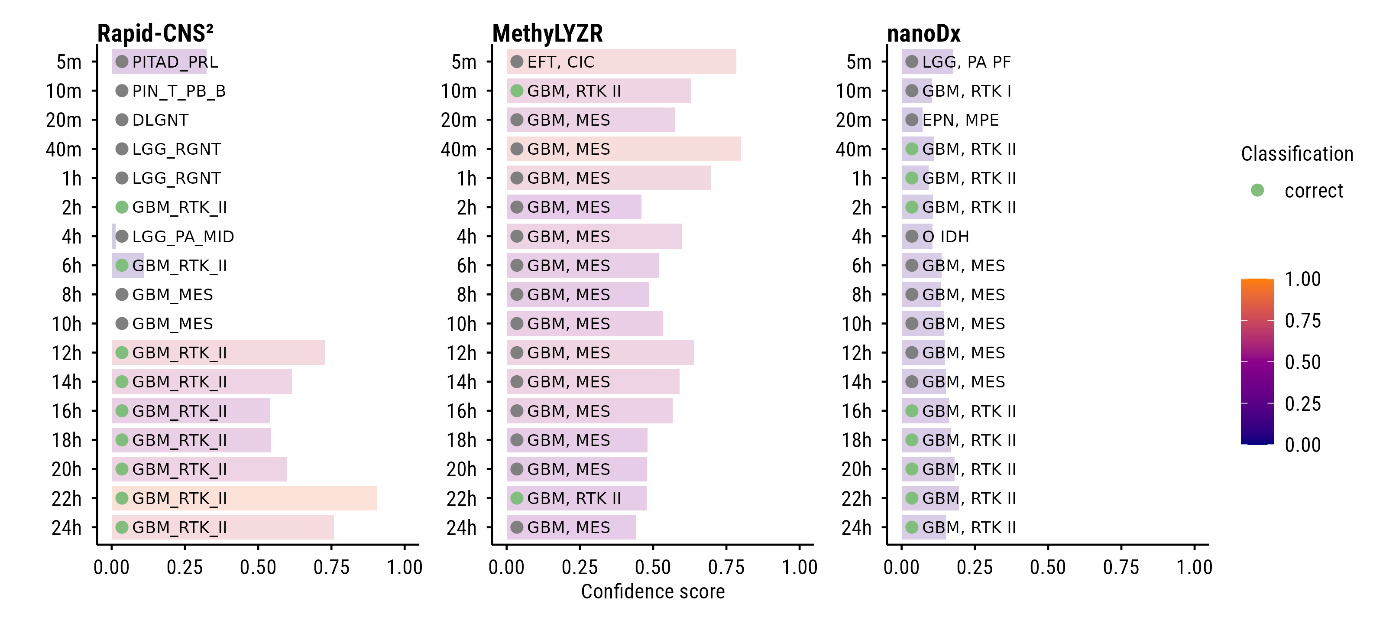


**Figure S2:** Detailed classification results over sequencing time for sample 16.


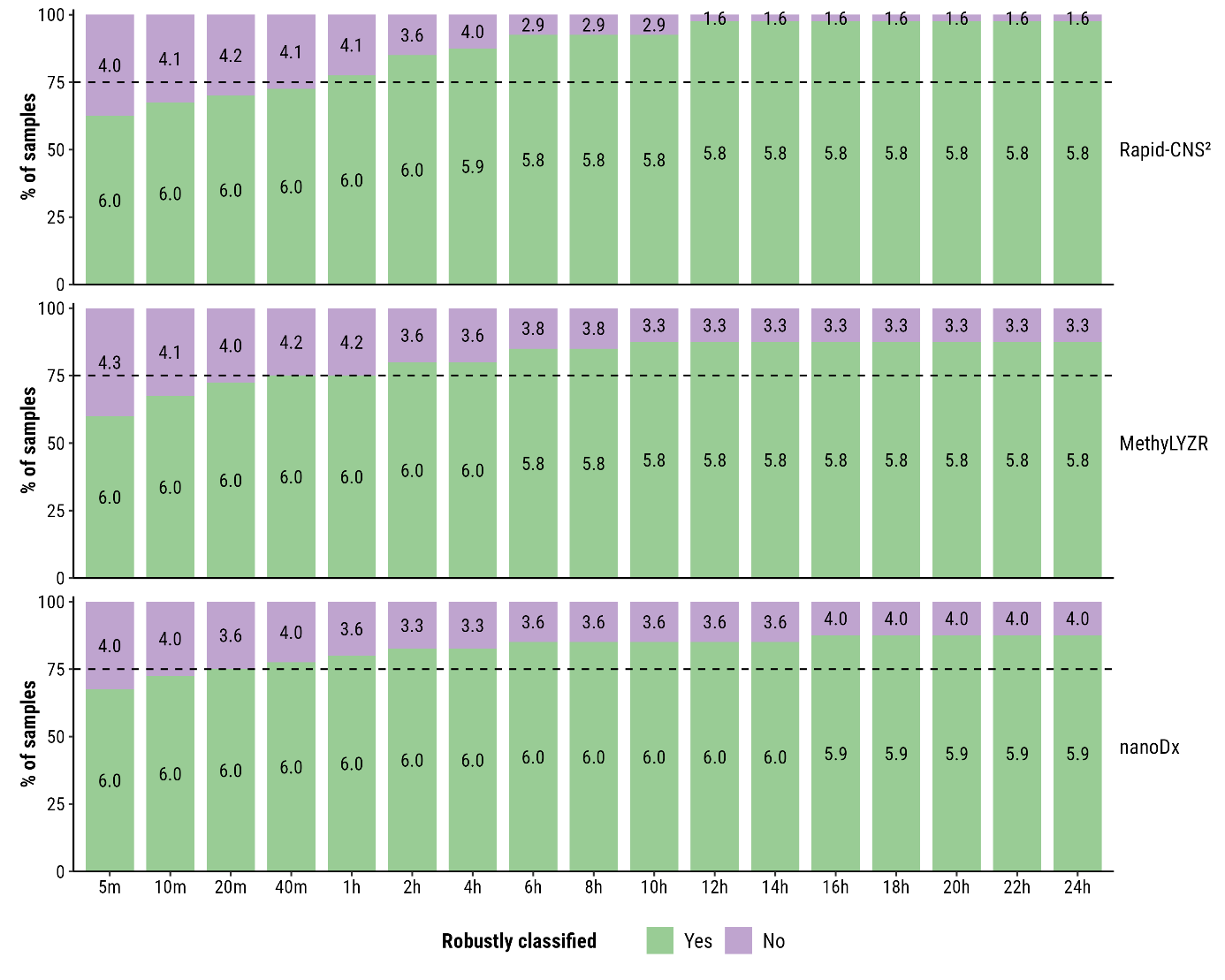


***Figure S3:*** Percentage of samples that were robustly classified by Rapid-CNS^2^, MethyLYZR, and nanoDx across multiple sequencing time points. Labels on bars indicate median DIN values.


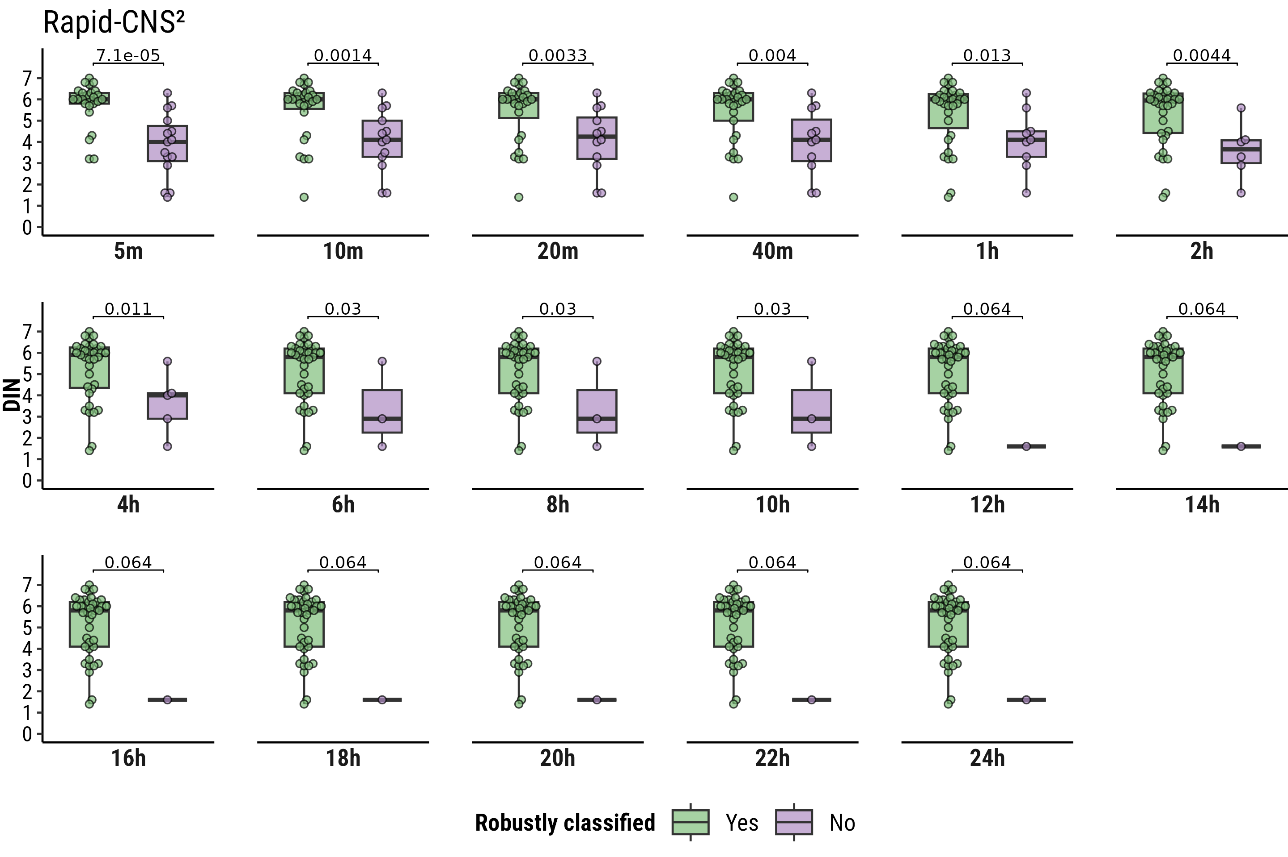


**Figure S4:** Comparison of DIN values between samples robustly classified by Rapid-CNS^2^ and samples not robustly classified across multiple sequencing time points. Statistical significance was determined using a one-sided, unpaired Wilcoxon rank-sum test.


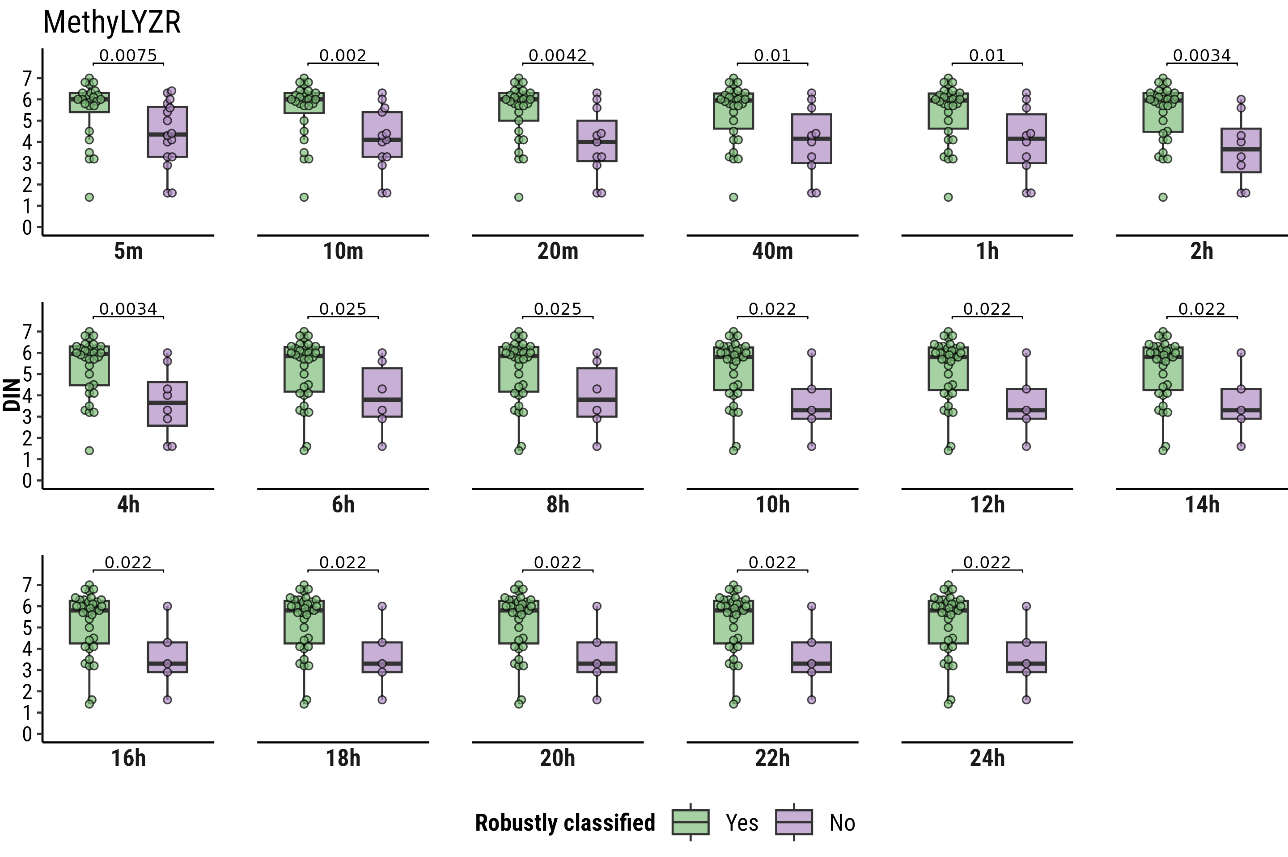


**Figure S5:** Comparison of DIN values between samples robustly classified by MethyLYZR and samples not robustly classified across multiple sequencing time points. Statistical significance was determined using a one-sided, unpaired Wilcoxon rank-sum test.


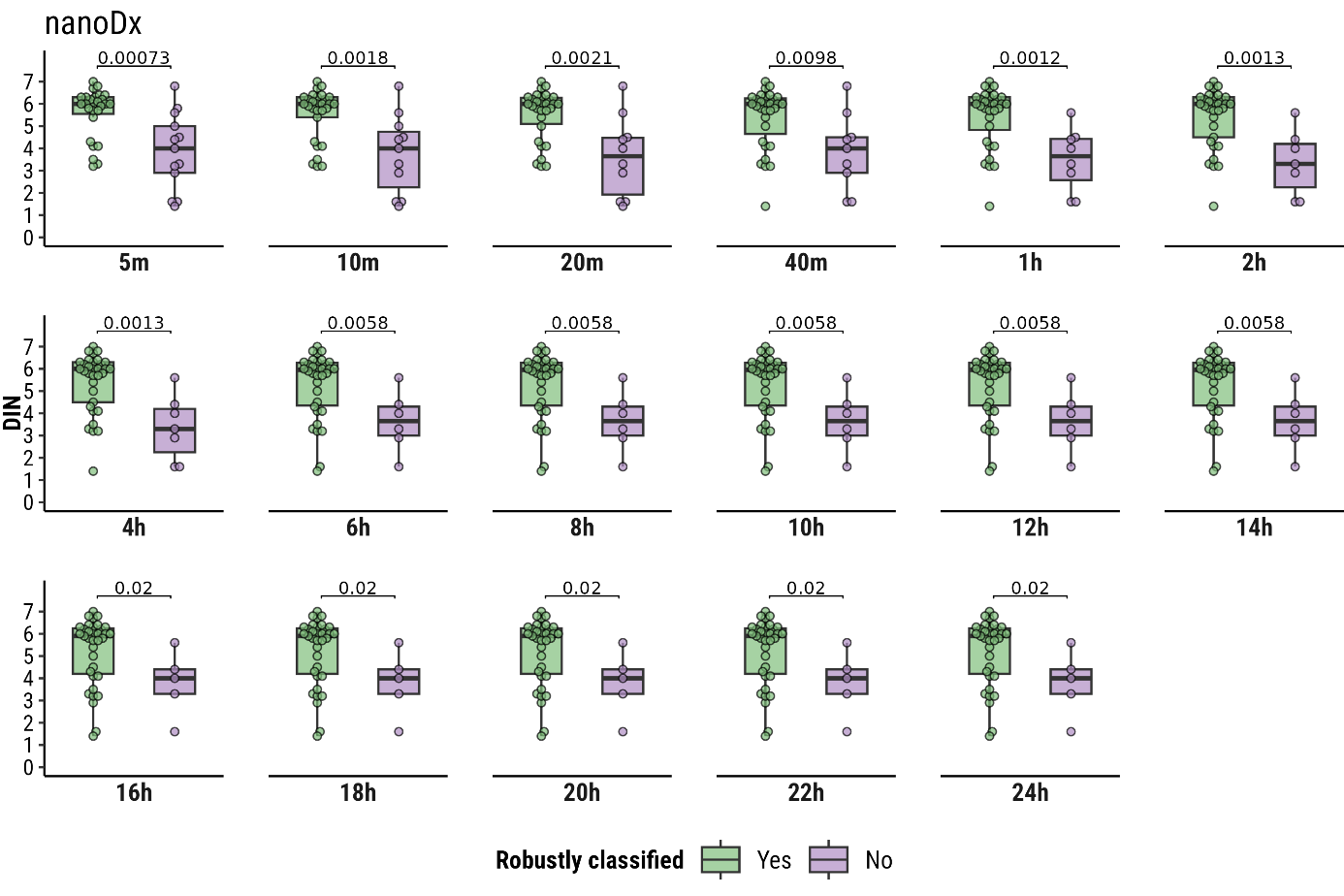


**Figure S6:** Comparison of DIN values between samples robustly classified by nanoDx and samples not robustly classified across multiple sequencing time points. Statistical significance was determined using a one-sided, unpaired Wilcoxon rank-sum test.


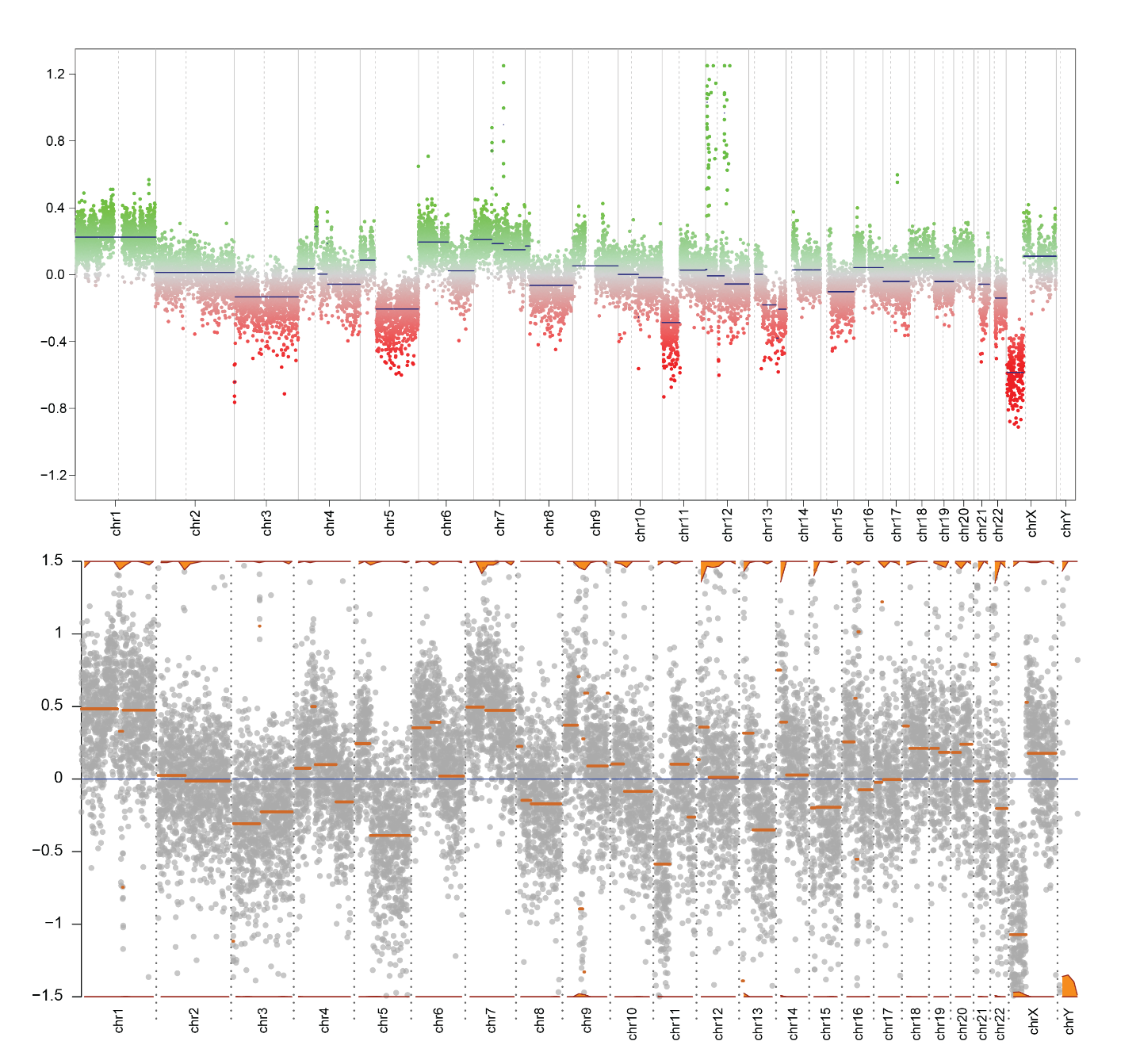


**Figure S7:** Visual comparison of the CNV profile for sample 6. Profiles were generated from EPIC data (upper plot) and Oxford Nanopore sequencing (lower plot). The Average Aitchison distance was 2.02. The DIN value for this sample was 5.8.


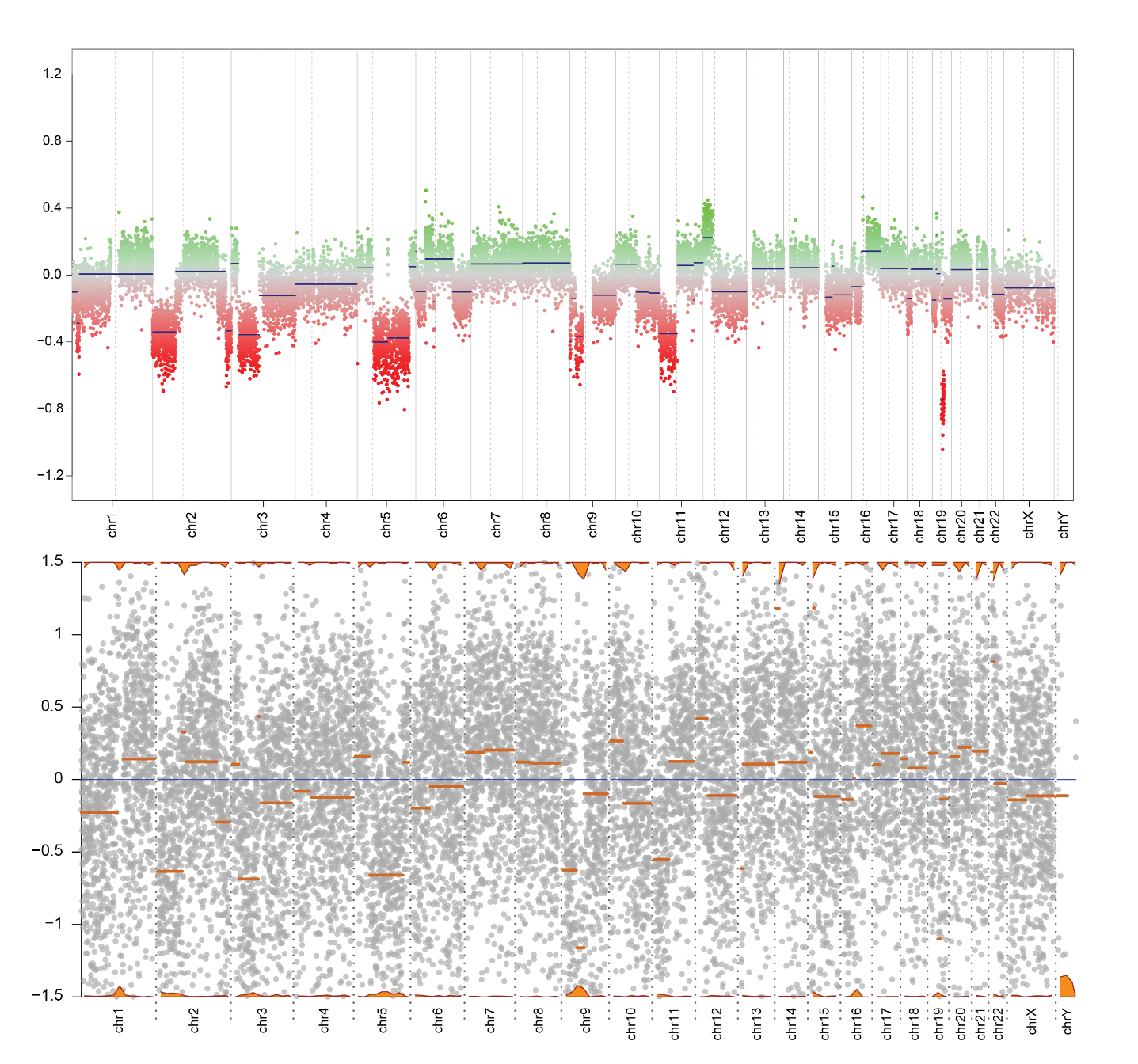


**Figure S8:** Visual comparison of the CNV profile for sample 8. Profiles were generated from EPIC data (upper plot) and Oxford Nanopore sequencing (lower plot). The average Aitchison distance was 3.41. The DIN value for this sample was 3.3.


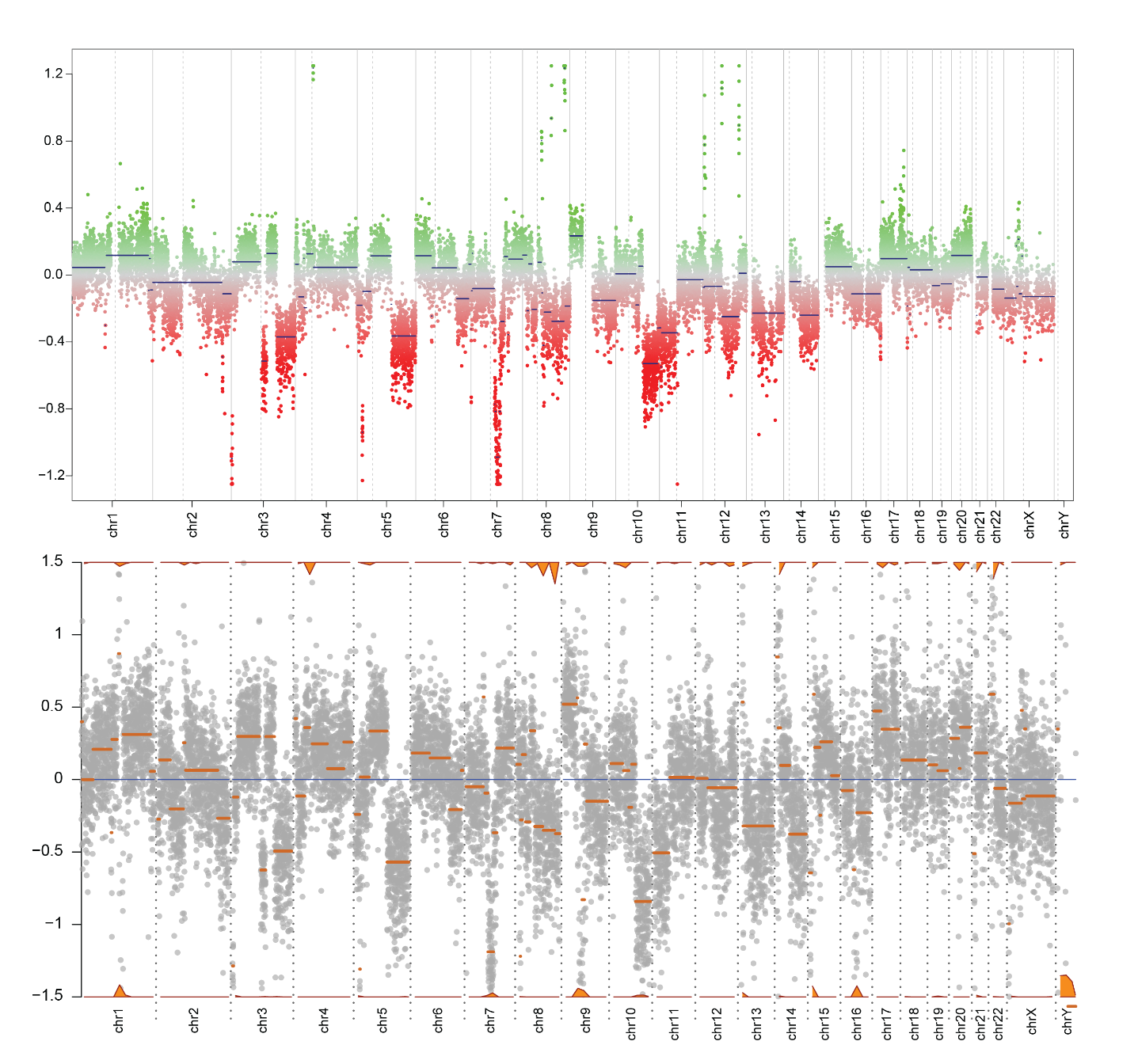


**Figure S9:** Visual comparison of the CNV profile for sample 10. Profiles were generated from EPIC data (upper plot) and Oxford Nanopore sequencing (lower plot). The average Aitchison distance was 3.12. The DIN value for this sample was 3.5.


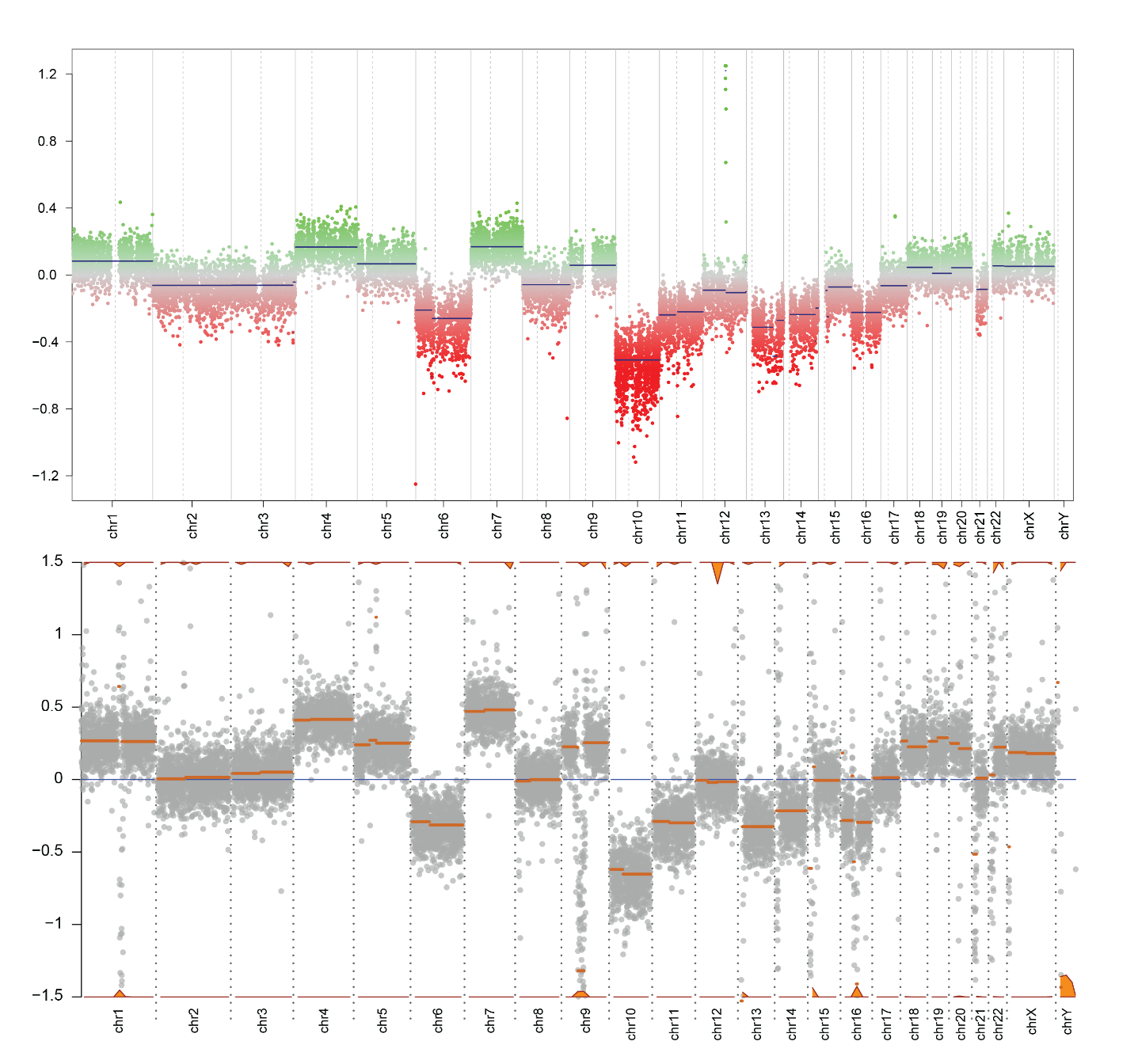


**Figure S10:** Visual comparison of the CNV profile for sample 13. Profiles were generated from EPIC data (upper plot) and Oxford Nanopore sequencing (lower plot). The average Aitchison distance was 3.92. The DIN value for this sample was 5.6.


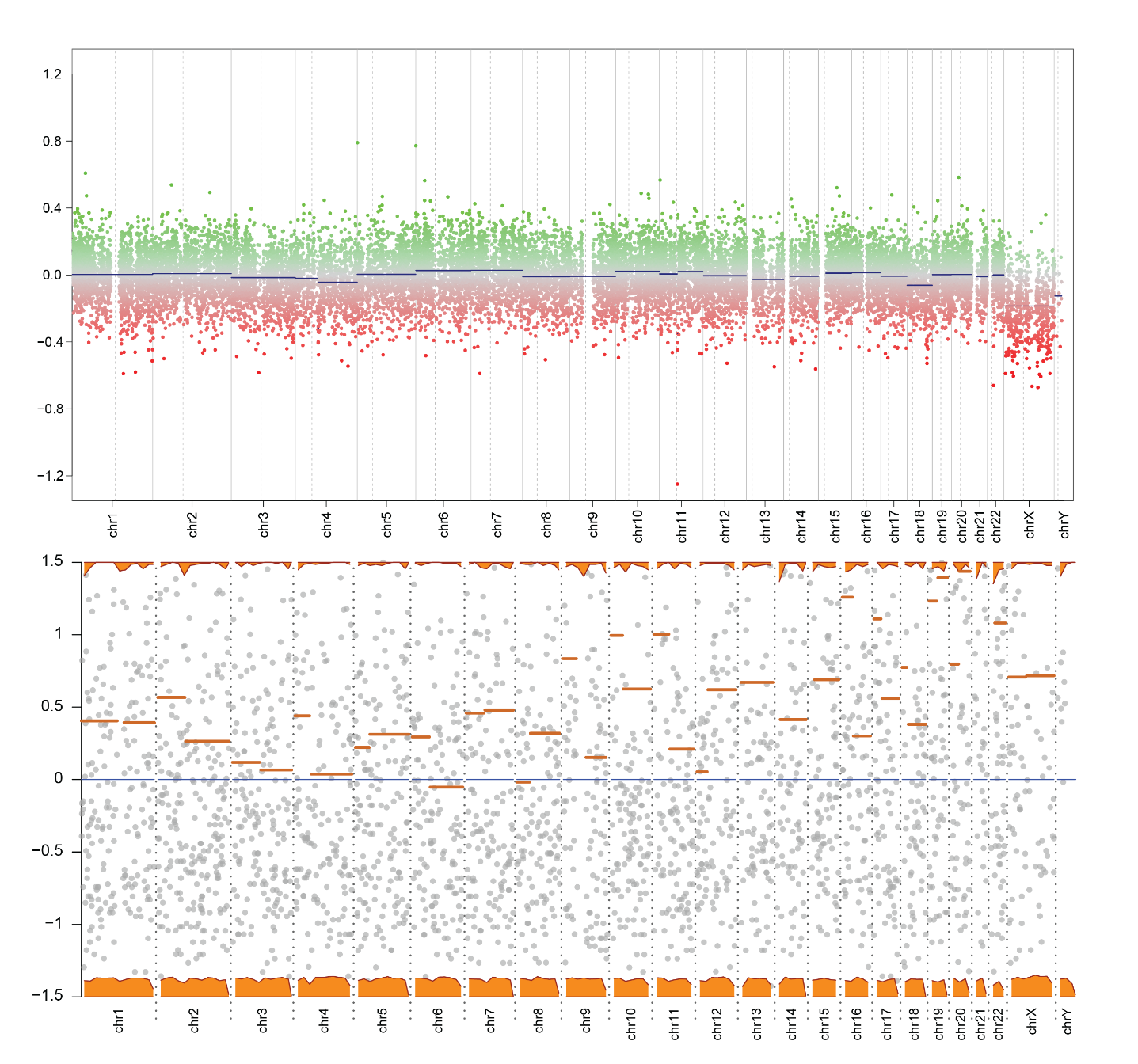


**Figure S11:** Visual comparison of the CNV profile for sample 21. Profiles were generated from EPIC data (upper plot) and Oxford Nanopore sequencing (lower plot). The average Aitchison distance was 5.52. The DIN value for this sample was 1.6.


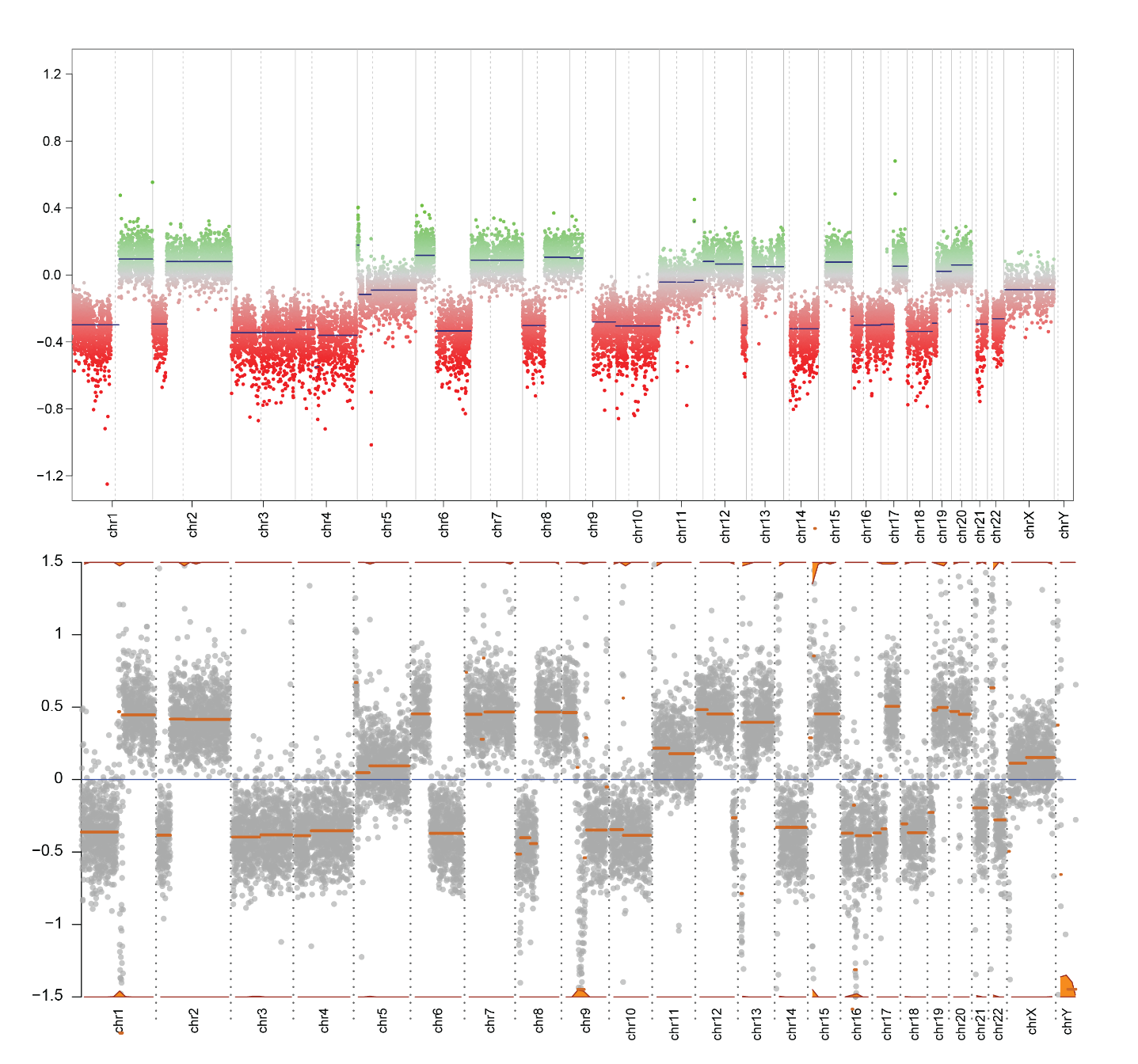


**Figure S12:** Visual comparison of the CNV profile for sample 29. Profiles were generated from EPIC data (upper plot) and Oxford Nanopore sequencing (lower plot). The average Aitchison distance was 4.18. The DIN value for this sample was 4.3.


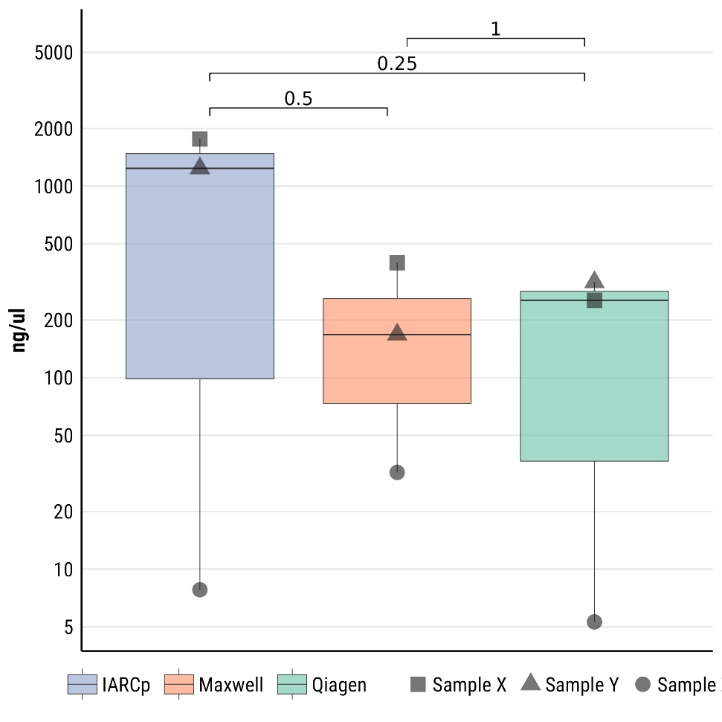


**Figure S13:** Comparison of DNA yields obtained using three extraction protocols: IARCp, Maxwell, and Qiagen. Differences between protocols were evaluated using a one-sided, paired Wilcoxon rank-sum test.
